# Is There a Link Between Nutrition, Genetics, and Cardiovascular Disease?

**DOI:** 10.3390/jcdd7030033

**Published:** 2020-08-27

**Authors:** Marwan El Ghoch, Said El Shamieh

**Affiliations:** 1 Department of Nutrition and Dietetics, Faculty of Health Sciences, Beirut Arab University, P.O. Box 11-5020 Riad El Solh, Beirut 11072809, Lebanon; 2 Department of Medical Laboratory Technology, Faculty of Health Sciences, Beirut Arab University, P.O. Box 11-5020 Riad El Solh, Beirut 11072809, Lebanon; s.elshamieh@bau.edu.lb

Cardiovascular diseases (CVDs) are a group of disorders that mainly include coronary, cerebrovascular and rheumatic heart diseases [1]. CVDs are the primary cause of death worldwide, and genetic and environmental factors seem to play a determinant role in this [1]. In fact, cardiovascular diseases are strongly associated with certain lifestyle factors (i.e., diet and physical inactivity) [2,3] and nutrition-related diseases (i.e., obesity and type 2 diabetes) [4]. Moreover, genome-wide association studies have also identified numerous genomic loci that determine susceptibility to cardiovascular events [5]. Therefore, nutrition and genetics seem to interact in predisposing an individual to cardiovascular diseases [6]. The Special Issue “Nutrition, Genetics, and Cardiovascular Disease” of the *Journal of Cardiovascular Development and Disease* provided a platform for the presentation of recent advances in knowledge relating to nutrition, genetics and cardiovascular disease, from diverse scientific disciplines, and it included four original articles, one narrative review and one systematic review and meta-analysis. 

The first original article investigated MT-CYB mutations in acute rheumatic fever and rheumatic heart diseases among Senegalese patients. The authors of this study found a narrow link between MT-CYB mutations and acute rheumatic fever and its complications, i.e., rheumatic heart diseases [7]. In the second original study, conducted in Uruguay on two separate cohorts (children, n = 682; adolescents, n = 340), the authors tested potential associations between anthropometric parameters (i.e., weight, height and body mass index (BMI)) in early life stages and the state of the cardiovascular system in early childhood at the beginning of adulthood [8]. The authors found that the current z-BMI showed the greatest capacity to explain variations in cardiovascular properties at 6 and 18 years. However, body size at birth showed no association with arterial properties at 6 or 18 years of age [8].

In the third original study, conducted in Saudi Arabia, the authors tested differences in dietary patterns (expressed in terms of adherence to the “Healthy Saudi” dietary guidelines) between two groups of males: CVD group (N = 40) and non-CVD group (N = 40) [9]. The authors found higher adherence scores for fruit, olive oil and non-alcoholic beer in the non-CVD patients [9]. The fourth original study, conducted on 460 healthy Lebanese adults from the general population, focused on detecting the association between the polymorphism rs2569190A > G in CD14 and CVD risk factors such as hypercholesterolemia and hypertension [10]. The authors found no significant association with hypertension. However, rs2569190G in CD14 was found to be associated with a higher risk of developing hypercholesterolemia among the Lebanese population [10].

On the other hand, the first systematic review that was conducted in Lebanon using the “Preferred Reporting Items for Systematic Reviews and Meta-Analyses” (PRISMA) guidelines in focused on clarifying whether hookah smoking is associated with a higher risk of obesity among the general population [11]. All the five included studies reported that hookah smoking increases the risk of obesity among all ages and in both genders, and this was confirmed by the meta-analysis [11]. Finally, in the narrative review of the renin angiotensin system (RAS) (known to be an endocrine system involved in blood-pressure regulation and body electrolyte balance) [12], the authors described the new components of RAS, their tissue-specific expression and their alterations under pathological conditions, which may facilitate development of more specific and personalized treatments [12].

In conclusion, the findings of the original articles and reviews of this Special Issue highlight certain nutritional and genetic features that seem to play an important role in CVDs.
